# Normalization and expression changes in predefined *sets *of proteins using 2D gel electrophoresis: A proteomic study of L-DOPA induced dyskinesia in an animal model of Parkinson's disease using DIGE

**DOI:** 10.1186/1471-2105-7-475

**Published:** 2006-10-26

**Authors:** Kim Kultima, Birger Scholz, Henrik Alm, Karl Sköld, Marcus Svensson, Alan R Crossman, Erwan Bezard, Per E Andrén, Ingrid Lönnstedt

**Affiliations:** 1 Department of Pharmaceutical Biosciences, Division of Toxicology, Uppsala University, BMC, Box 594, SE-75124 Uppsala, Sweden; 2 Laboratory for Biological and Medical Mass Spectrometry, Uppsala University, Box 583, SE-75123 Uppsala, Sweden; 3 Department of Pharmaceutical Biosciences, Uppsala University, BMC, Box 583, SE-75123 Uppsala, Sweden; 4 Faculty of Life Sciences, The University of Manchester, UK; 5 CNRS UMR 5543, University Victor Segalen, Bordeaux, France; 6 Department of Mathematics, Uppsala University, Box 480, SE-75106 Uppsala, Sweden

## Abstract

**Background:**

Two-Dimensional Difference In Gel Electrophoresis (2D-DIGE) is a powerful tool for measuring differences in protein expression between samples or conditions. However, to remove systematic variability within and between gels the data has to be normalized.

In this study we examined the ability of four existing and four novel normalization methods to remove systematic bias in data produced with 2D-DIGE. We also propose a modification of an existing method where the statistical framework determines whether a *set *of proteins shows an association with the predefined phenotypes of interest. This method was applied to our data generated from a monkey model (*Macaca fascicularis*) of Parkinson's disease.

**Results:**

Using 2D-DIGE we analysed the protein content of the striatum from 6 control and 21 MPTP-treated monkeys, with or without de novo or long-term L-DOPA administration.

There was an intensity and spatial bias in the data of all the gels examined in this study. Only two of the eight normalization methods evaluated ('2D loess+scale' and 'SC-2D+quantile') successfully removed both the intensity and spatial bias. In 'SC-2D+quantile' we extended the commonly used loess normalization method against dye bias in two-channel microarray systems to suit systems with three or more channels.

Further, by using the proposed method, Differential Expression in Predefined Proteins *Sets *(DEPPS), several *sets *of proteins associated with the priming effects of L-DOPA in the striatum in parkinsonian animals were identified. Three of these *sets *are proteins involved in energy metabolism and one *set *involved proteins which are part of the microtubule cytoskeleton.

**Conclusion:**

Comparison of the different methods leads to a series of methodological recommendations for the normalization and the analysis of data, depending on the experimental design. Due to the nature of 2D-DIGE data we recommend that the p-values obtained in significance tests should be used as rankings only. Individual proteins may be interesting as such, but by studying *sets *of proteins the interpretation of the results are probably more accurate and biologically informative.

## Background

Proteomic techniques are important tools for studying the mechanisms of a disease, pinpointing new therapeutic targets or finding potential biomarkers. The field of proteomics is ever expanding and today there are several techniques available for protein separation, both gel based and non-gel based. Traditionally two-dimensional polyacrylamide gel electrophoresis (2D-PAGE) has been the technique used for protein separation. In 2D-PAGE proteins first undergo isoelectric focusing (IEF) based on their net charge, then an orthogonal second dimension is applied to further separate proteins based on their molecular weight, in the presence of denaturing conditions. In this way it is possible to resolve several thousand proteins in a single sample. 2D-PAGE mainly produces data which enables the investigator to determine whether a particular protein shows an increase or decrease when comparing two different conditions e.g. a diseased state compared to a non-diseased state. The limited dynamic range and poor reproducibility between gels has been of major concern with traditional 2D-PAGE experiments.

The task of detecting changes in protein expression has recently been facilitated by the introduction of difference in gel electrophoresis (DIGE)[1,2]. Gels using the 2D-DIGE method usually contain three samples labeled with three distinct fluorescent dyes, Cy2, Cy3 and Cy5. The Cy2 dye is typically used to label an internal standard which is a mix of all samples in the experiment and the other two dyes are usually used to label two biological samples of interest. The strength of the internal standard is to help the mapping of spots/proteins between gels and thus make the different gels more comparable. The internal standard is also used in some methods for normalization within and between gels. The 2D-DIGE has been commercialized through the Ettan DIGE System of Amersham Biosciences (now GE Healthcare).

Fluorescent dyes, most commonly Cy3 and Cy5, have been used extensively in gene expression microarray technologies to measure differences in gene expression. Using cDNA microarrays there is a need for proper normalization in order to remove systematic variation, within and sometimes between arrays, and a need for proper test statistics to exploit the information across genes [3-8].

Since the data generated from 2D-DIGE experiments exhibits similar characteristics to that obtained from cDNA gene expression microarrays, some methods have evolved for normalization of data produced with 2D-DIGE based on methods in gene expression analysis [9,10]. These methods focus on the intensity bias within and between gels, paying little attention to spatial bias within gels. Spatial bias is known to be a source of variation in gene expression microarrays [8].

In a gel set using 2D-DIGE we examined spatial and intensity bias removal by eight different normalization methods. The commercial software available from GE Healthcare (DeCyder) provides two of the methods, two of the methods have been used on 2D-DIGE data before [9,10], two methods have not been used on 2D-DIGE data before, but have been used on gene expression microarray data [3,8] and the final two are single channel analysis approaches which have not been used on 2D-DIGE data previously.

Most 2D-DIGE data analysis has focused on finding single proteins with a changed expression between two different conditions and tested for significant differences between the means or medians in the different groups. To our knowledge the most common approaches are those of two-sample t-tests, their extensions within the scope of analysis of variance (ANOVA) [11] and Generalized Linear Models (GLM) [12], and moderated t-statistics [6,9,13].

The issue of significance level correction in multiple hypothesis tests has been extensively discussed in the field of gene expression microarrays. In proteomics studies, we and others have used methods correcting the observed significance levels using false discovery rate (FDR) [9,13]. There are several similarities between data obtained from gene expression and proteins (2D-DIGE), but there are also aspects of the data that have to be taken into account. To a single protein there may be different chemical moieties attached or removed by various enzymes, also known as post-translational modification (PTM). This causes a change in the protein mass and charge. As a result a protein originating from the same gene-product but with different modifications may be found in several different positions in the gels. A treatment effect on a protein may therefore cause a change of level of the unmodified protein and/or a change in level for a certain PTM-protein. It is difficult to know whether these actions are co-regulated or not, but as a consequence the use of FDR may be inappropriate since it assumes independent observations. Instead we propose the use of an alternative method which produces cut-off levels on the basis of permutation tests.

Although single differentially expressed spots/proteins can be informative, we are primarily interested in the activity profiles of *sets *of functionally related proteins. Therefore, we also perform re-sampling-based tests on predefined *sets *of proteins. Our protein set test is in line with gene set tests suggested by Subramanian [14] and Tian [15]. We adapted their method to suit protein data and have named it Differential Expression in Predefined Protein *Sets *(DEPPS).

Parkinson's disease is a progressive neurodegenerative disorder which is characterized by the degeneration of dopaminergic neurons of the substantia nigra, causing a reduction in striatal dopamine content. Dopamine replacement by L-DOPA is the most common treatment resulting in an initial positive symptomatic response. Unfortunately, long-term L-DOPA therapy is associated with the development of motor complications such as dyskinesia. After 4–6 years of L-DOPA treatment approximately 40% of patients have developed dyskinesia [16]. Once exposed to L-DOPA therapy, some patients are 'primed' and some will eventually develop dyskinesia even if switched to a drug that in itself does not induce dyskinesia when administered de novo [17]. Dyskinesia contributes to the disability experienced by patients and it is therefore of great importance to understand the mechanisms of L-DOPA induced dyskinesia.

In this report we analyze the protein content in the striatum from the gold-standard animal (*Macaca fascicularis*) model of Parkinson's disease with and without de novo or long-term L-DOPA treatment. The group with long-term L-DOPA treatment displayed dyskinetic symptoms. We examine the ability of eight normalization methods to remove the intensity and spatial bias found in our data produced with 2D-DIGE. Four of the methods have previously been used for data generated from 2D-DIGE and four of them are novel. We also discuss existing methods for producing cut-off levels for finding differentially expressed spots/proteins and propose an alternative method based on permutation tests. Finally we use the method proposed in this study, DEPPS, to provide insights on the priming effects of L-DOPA in the striatum in parkinsonian animals.

## Results

We have used 2D-DIGE to study the difference in protein expression in the striatum of 27 animals which received four different administration regimens. Six of the animals were used as controls (Ctl), five were administered MPTP only (Mptp), six animals were administered MPTP and then a single dose of L-DOPA (Ldopa) and ten animals were first administered MPTP then long-term treatment with L-DOPA until displaying dyskinesia (Dysk). One of the control samples were used twice, resulting in a total of 28 samples which were compared on 14 2D-DIGE gels.

All gel images were analyzed using the DeCyder software v5.02 (GE Healthcare). On average 1126 ± 64 (standard error of mean) spots were identified on the gels. As gel number four displayed the most spots (1851), it was designated as the master gel for matching purposes. For evaluating the different normalization methods, we included spots from each gel that were also found in the master gel. When comparing the four experimental groups we only included spots for which we had enough observations to estimate all possible comparisons between treatment groups, and for which the degrees of freedom in the linear model were at least eleven. The quality of all these spots was examined manually and subsequently 1211 spots were used in the parameter estimation.

### Evaluation of bias removal by different normalization methods

We have studied eight different normalization methods. Two of the methods are provided with the DeCyder software ('DeCyder no pool' and 'DeCyder pool'), two have been published earlier in the literature ('Fodor' and 'Kreil') [9,10], two are known from two-channel cDNA expression array data but have now been adapted to suit 2D-DIGE data ('loess+scale' and '2D loess+scale') and the final two are a separate channel analysis approach that have not been used on 2D-DIGE data before ('SC-quantile' and 'SC-2D+quantile'). For comparison, we have also included un-normalized data (raw data).

**Figure 1:**
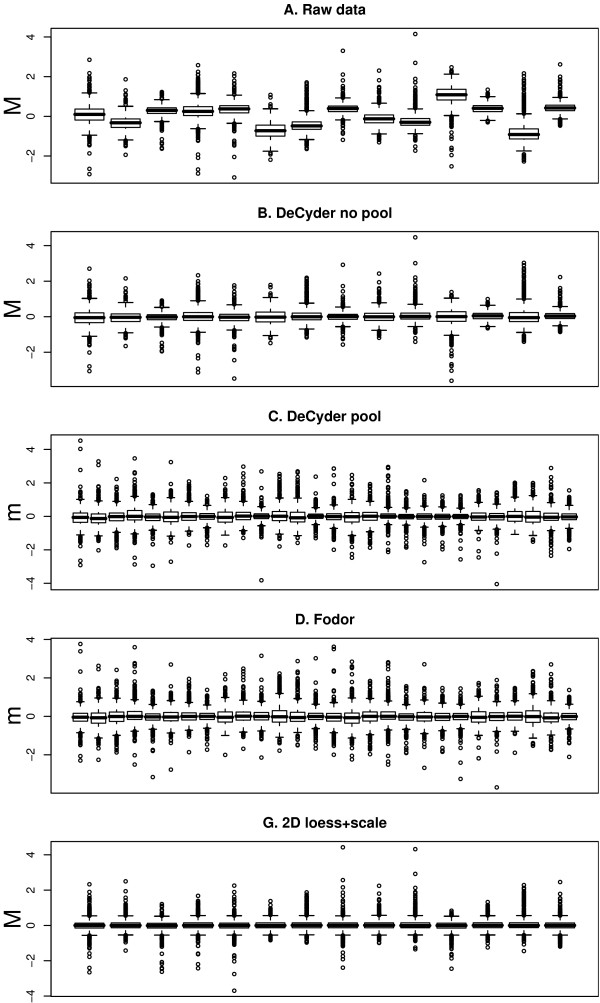
**Boxplots of M or m values**. Boxplots of the *M or m*-values for the 14 gels (A) before, and (B-E) after normalization using different methods. Gels are ordered (1–14) according to the experimental design in Table 1. For method A, B and E the *M*-values are calculated as log_2_*Cy5*/*Cy3 *and for method C and D the *m*-values are calculated as log_2_*Cy5*/*Cy2 *and log_2_*Cy3*/*Cy2*. For method C and D this results in two boxplots for each gel. After optimal normalization the *M *or *m*-values should average to zero and have approximately the same variance (equally high boxes) in all gels. The methods which are not illustrated in the figure show an impeccable pattern similar to that of (E).

**Figure 2:**
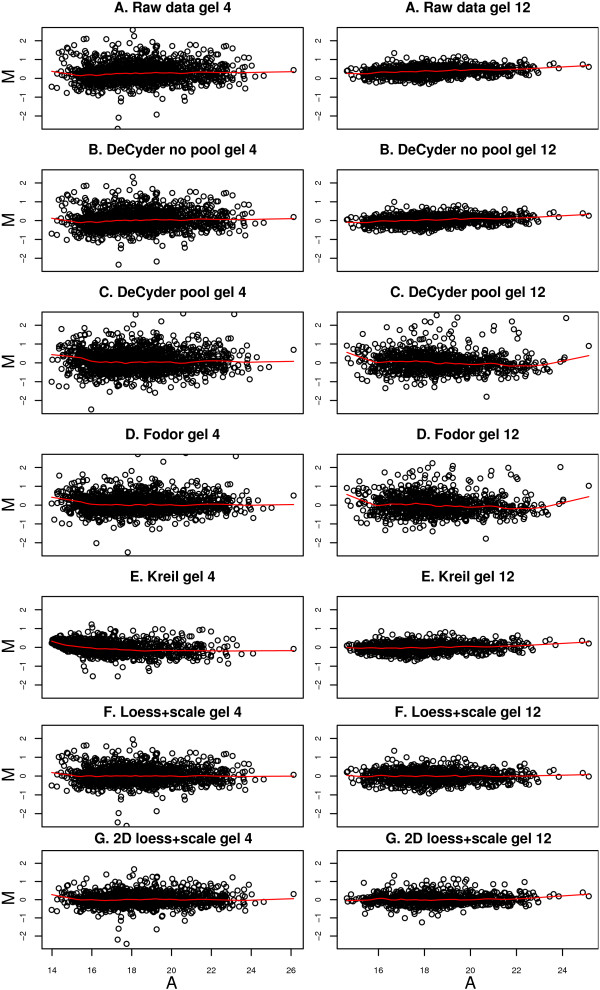
**MA plots for gel 4 and 12**. MA plots for gel 4 and 12 (A) before, and (B-E) after normalization using different methods. The average log_2 _intensity, *A*, is calculated as (log_2_*Cy5*+log_2_*Cy3*)/2. For method A, B, E, F and G the *M*-values are calculated as log_2_*Cy5*/*Cy3 *and for method C and D the *M*-values are calculated as log_2_(*Cy5*/*Cy2*)/(*Cy3*/*Cy2*). The red line in each plot shows the Lowess smoothing of the entire data in the plot and should ideally be a straight line on zero. The method by Kreil *et al *[10] gives intensity dependant bias in the low range in gel 4 and when the *M *values are calculated via the pool channel (Cy2, method C and D) the data tend to be more variable.

**Figure 3:**
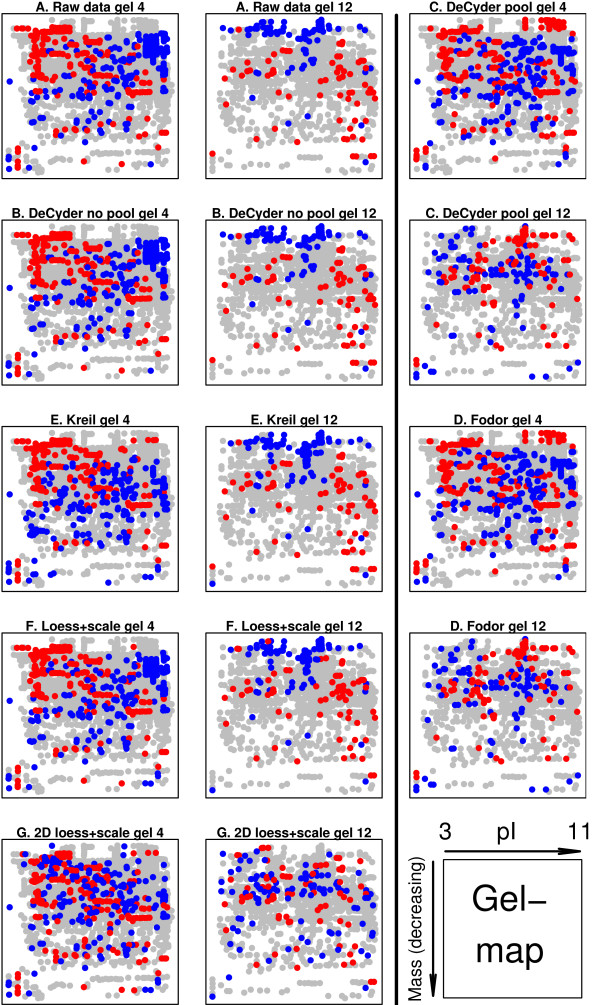
**2D-M plot for gel 4 and 12**. Spatial gel plots for gel 4 and 12 (A) before, and (B-E) after normalization using different methods. A reconstructed gel image with increasing isoelectric point (pI) (left-right) and decreasing protein mass (top-down). For method A, B, E, F and G the *M*-values are calculated as log_2_*Cy5*/*Cy3 *and for method C and D the *M*-values are calculated as log_2_(*Cy5*/*Cy2*)/(*Cy3*/*Cy2*). The ten percent highest and lowest *M*-values in each gel are color coded red and blue respectively. For several of the normalization methods the spatial bias remains.

When the raw data was analyzed using only Cy5 and Cy3 intensities, significant dye bias was found in several of the gels (Figure 1A). This dye bias was intensity dependant in some of the gels (Figure 2A). Each of the 14 gels exhibited spatial bias, irrespective of the treatment group identity of the samples. For instance, in the top right corner of gel four there were generally higher Cy3 intensities compared to Cy5. The same applied for the higher mass regions (top) of gel twelve (Figure 3A).

The 'DeCyder no pool' successfully removed dye-specific differences (Figure 1B&2B). The same applied to the 'Loess+scale' method (data not shown and Figure 2F). The 'Kreil' method introduced bias in the lower intensity range in some of the gels (Figure 2E, gel 4). However, none of three methods removed the spatial bias seen in the raw data (Figure 3B,E &3F).

Two of the pool-based normalization methods ('DeCyder pool' and 'Fodor') removed the dye-specific differences, but spatial bias remained (Figure 1C&1D and Figure 3C&3D). Because the Cy5/Cy3 logratios are calculated via the pool channel (Cy2) the spatial pattern is different compared to the methods not using the pool.

Comparing the two normalization methods included in the DeCyder software ('DeCyder pool' and 'DeCyder no pool') where the fold changes are calculated with or without the pool channel (Cy2), the variability is larger when the pool channel is included (Figure 2B&2C). This is a natural consequence when two signals (Cy3 and Cy5) are compared via a third signal (Cy2) rather than directly. The two methods using the pool channel, 'DeCyder pool' and 'Fodor', produced the largest variability in fold changes compared to all other methods, including the raw data as can be seen in Figure 2C&2D.

When using the proposed '2D loess+scale' method the spatial and intensity dependant dye bias were successfully removed (Figure 2G&3G). From this observation we suggest that the intensity dependant bias often found in 2D-DIGE data may be completely or partly dependant on spatial bias. To adjust the scales of the logratio distributions the 2D loess adjustment was followed by between gel scale normalization (Figure 1E).

**Figure 4:**
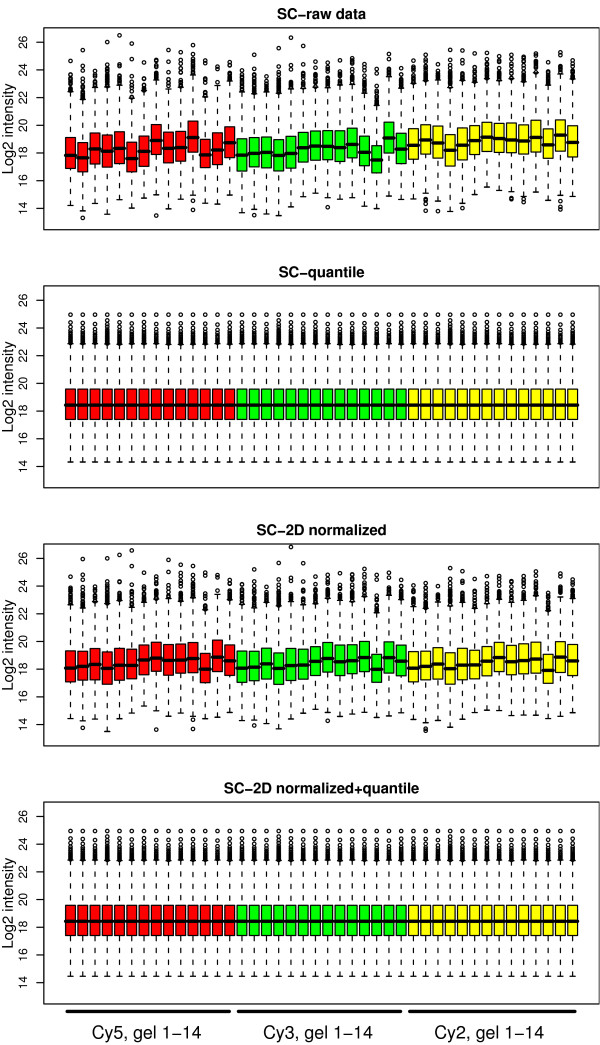
**Boxplots of intensity values**. Boxplots of the log_2 _intensity values for the 14 gels before and after normalization using different methods. Red boxes (1–14) are Cy5, green boxes (15–28) are Cy3 and yellow boxes (29–42) are Cy2 intensities. The data for each dye is ordered gel 1–14. After optimal normalization the intensity values should have approximately the same average and empirical distribution (equally high boxes) in all gels.

**Figure 5:**
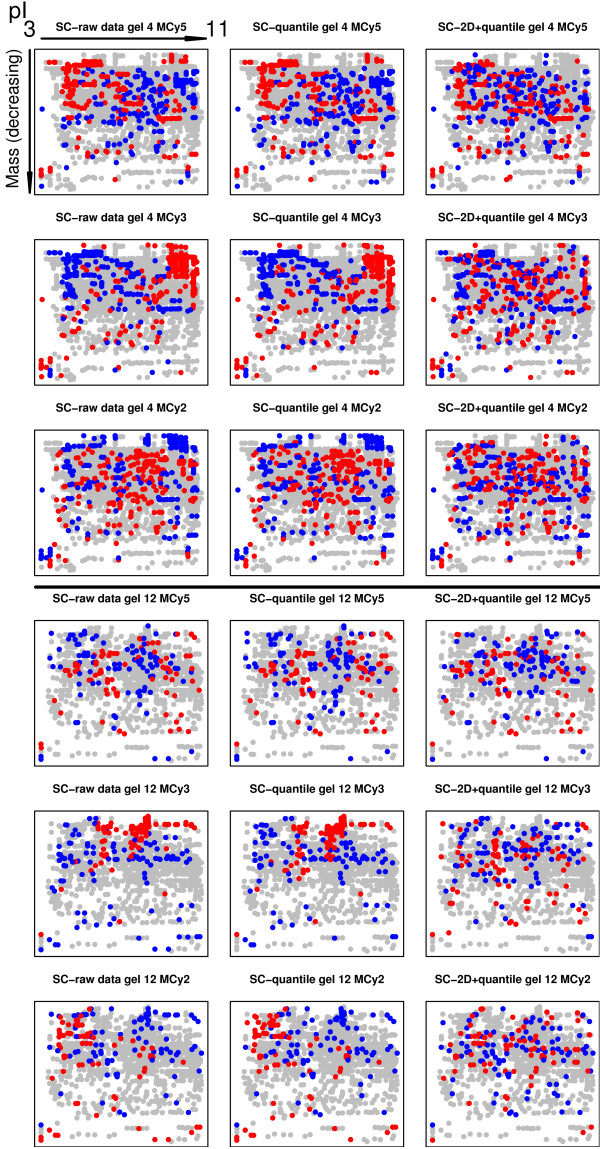
**2D-Intensity plots for gel 4 and 12**. Spatial gel plots of Cy5, Cy3 and Cy2 intensities versus the average intensity for gel 4 and 12 before, and after normalization using different methods. Each *MCy5*, *MCy*3 and *MCy*2 -spot value are calculated as the *Cy5*, *Cy3 *or *Cy2*-value divided by the average intensity *I*, (log_2_*Cy5*+ log_2_*Cy3*+ log_2_*Cy2*)/3. A reconstructed gel image for gel 4 and 12 with increasing pI (left-right) and decreasing protein mass (top-down). The ten percent highest and lowest (*MCy5*, *MCy*3 and *MCy*2) -spot values in each gel are color coded red and blue respectively. There is a significant spatial bias in the raw data and after 'SC-quantile' normalization, which is removed using the 'SC-2D+quantile' method.

In the single channel analysis, each of the three channels on the 14 gels had different mean intensity but the variance of the data was quite similar, as can be seen by the width of the boxes in Figure 4. Quantile normalization ('SC-quantile') forces each of the channels to have the same mean intensity and range, but the spatial bias is similar to that seen in raw data (Figure 4&5).

For the method 'SC-2D+quantile' when spatial location normalization is applied to each of the three intensity channels, the spatial bias is removed, but there are still differences between the intensity means, therefore quantile normalization is also applied (Figure 4&5).

**Figure 6:**
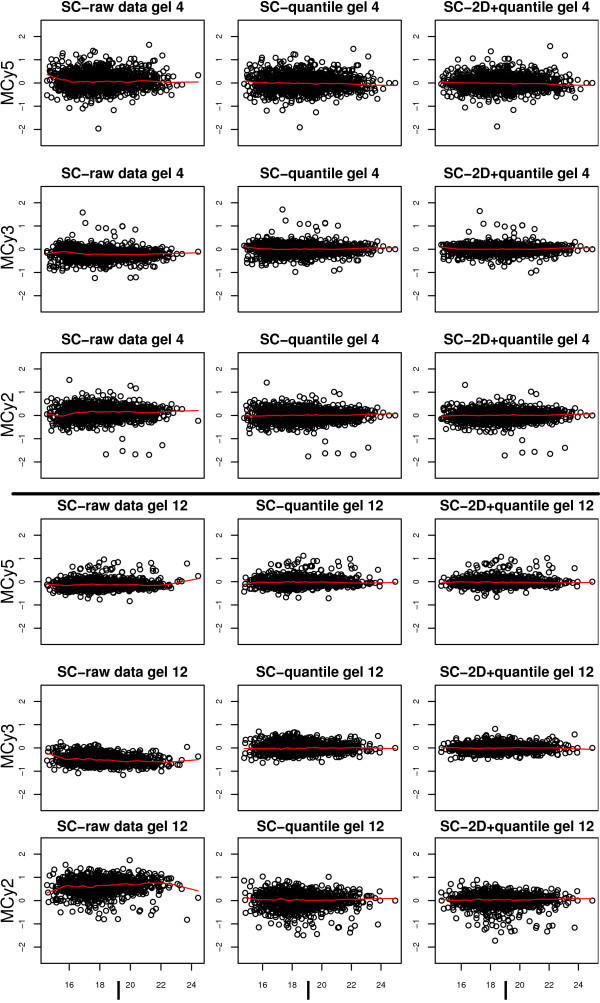
**3Dyes-I plots for gel 4 and 12**. 3Dyes-I plots for gel 4 and 12 before and after normalization using different methods. Each *MCy5*, *MCy*3 and *MCy*2 -spot value are calculated as the *Cy5*, *Cy3 *or *Cy2*-value divided by the average intensity *I*, (log_2_*Cy5*+ log_2_*Cy3*+ log_2_*Cy2*)/3. The red line in each plot shows the Lowess smoothing of the entire data in the plot and should ideally be a straight line on zero. Both the 'SC-quantile' and 'SC-2D+quantile' methods remove intensity dependant bias. The values for extreme observations are more or less unaffected after spatial loess normalization.

Importantly the values of extreme observations are very similar for raw data and data after spatial location normalization (both for the two and three dye normalization methods). These extreme values may represent true biological differences between samples and should therefore only be slightly affected by the normalization (Figure 2&6).

### Effects of different normalizations

All normalization methods gave different results for the estimated spot expression differences between treatment groups.

After normalization the spots were ranked and plotted in a volcano plot. A volcano plot displays the measure of statistical significance of the change, lodsratio (for log-odds ratio) versus the fold changes. A high lodsratio indicates a higher chance of true differential expression compared to a low lodsratio (see also Methods: Parameter estimation). This method is widely used on data generated from gene expression arrays [5,6] and occasionally on 2D-DIGE data [13]. We calculated the lodsratios using the eBayes function in the Limma [6] R [18] package.

**Figure 7:**
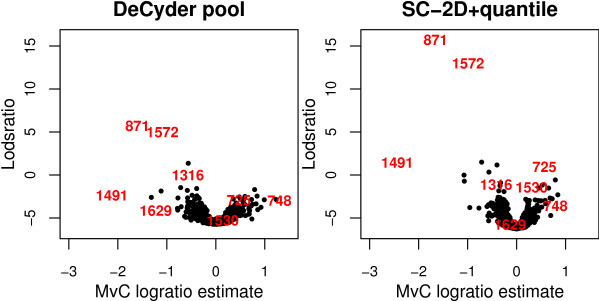
**Volcano plots comparing 'DeCyder pool' and'SC-2D+quantile'**. Volcano plots showing the lodsratio and *M*-values for the *MvC *estimate using the two different normalization methods 'DeCyder pool' and the 'SC-2D+quantile'. There are several spots with similar ranking, but there are also spots with great differences in the lodsratio ranking and *M*-values.

Figure 7 displays volcano plots comparing the experimental groups Mptp and Ctl (*MvC*) (see Methods: Parameter estimation) based on the method 'DeCyder pool' (which requires the use of the pool channel in the estimate of each Cy5 and Cy3 value, and assumes the two estimates to be independent) and 'SC-2D+quantile' (which requires the use of the pool channel in the linear model and assumes the three intensities from each gel to be correlated). Several spots showed similar results with both methods; the highlighted spots indexed 871 and 1572 both had high lodsratios compared to the rest of the spots. Several spots such as 725, 748, 1491 and 1316 showed minor differences in rankings and estimates. However there were also several spots such as 1530 and 1629 with very different rankings and effect estimates.

The standard errors of the spot expression estimates are generally larger using the 'DeCyder pool' normalization compared to the 'SC-2D+quantile' method. This is indicated by the width of the 'DeCyder pool' volcano plot compared to that of 'SC-2D+quantile' (Figure 7).

Although we can not prove which method provides the most accurate results for each particular spot, reduced standard errors of the spot expression estimates generally gives better results.

The treatment effects and rankings could have been estimated only using the Cy5 and Cy3 intensities (normalize data using '2D loess+scale' method) and the pool channel would have been excluded. In this study, where the effects of all four treatments were compared with each other and the original design was fairly skewed, the analysis gained by including the pool channel (normalize data using 'SC-2D+quantile' method), especially for those treatments with few or no direct comparisons. See also Discussion; The pool channel and parameter estimation.

### Protein classification

Before any comparisons were made between the different treatments, 317 spots were picked for identification. Of these, 252 proteins were successfully identified. Based on the criterions described above (see also Methods: Parameter estimation) the level of 231 proteins had been compared between the four experimental groups. For the *set *tests (see below) ten proteins were excluded from the analysis due to limited information available on biological function. The remaining 221 proteins were successfully classified into groups (*sets*) based on molecular function, their membership in protein families, involvement in biological processes and cellular localization. A total of 47 *sets *were identified.

### Results of parameter estimation and single differentially expressed spots

**Figure 8:**
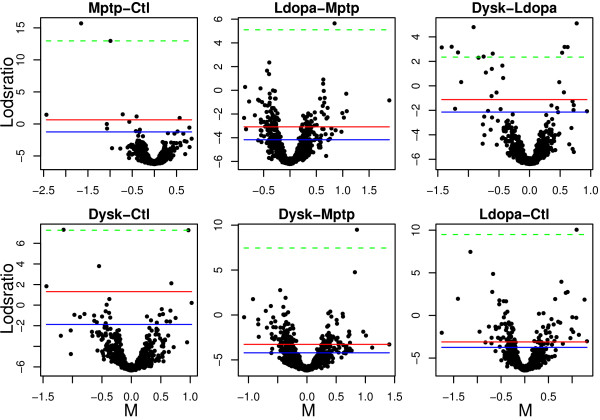
**Volcano plots for six comparisons of interest**. Volcano plots showing the lodsratios versus the effect estimates for six comparisons of interest. Cut-off levels in the lodsratio corresponding to FDR* equal to 0.30 (red), 0.50 (blue) and the minimum observed FDR* (dashed green) are plotted in each volcano plot.

Using the 'SC-2D+quantile' normalization and least squares estimates we calculated lodsratios for six contrasts of interest (see Methods: Parameter estimation). Figure 8 illustrates volcano plots of the comparisons between Mptp and Ctl (*MvC*), Ldopa and Mptp (*LvM*), Dysk and Ldopa (*DvL*), Dysk and Ctl (*DvC*), Dysk and Mptp (*DvM*) as well as that between Ldopa and Ctl (*LvC*).

Using a permutation test we estimated three lodsratio cut-offs for each comparison, corresponding to FDR* 0.3, 0.5 and the minimum observed FDR*. Using a FDR* value of 0.3 for all comparisons resulted in 203 unique spots above the lodsratios cut-offs whereas 57 of these spots have been successfully identified and classified into *sets*.

### Results of differential expression in predefined sets of proteins

The purpose in most 2D-DIGE gel experiments is to find single proteins found to be differentially expressed between e.g. two different treatments. However, we were primarily interested in the expression changes of predefined *sets *of proteins. It is anticipated that this also reduces the number of false positive results, which is a risk when looking at individual proteins on a large scale.

Based on the method presented by Subramanian *et al *[14] and Tian *et al *[15] we calculated significance levels (p-values) for *sets *of proteins that show association with the predefined phenotypes of interest.

The modified method used here, DEPPS, is based on ranking (using the lodsratio) for all the spots in each *set *of interest. An advantage with this kind of approach is that it uses the whole ranking list of spots/proteins and not only those above a certain lodsratio cut-off and/or fold change.

A p-value for each protein *set *was assessed by comparing the result from the true comparison to those from the permutations of gel numbers (10 000 permutations), (see Methods; Differential expression in predefined sets of proteins).

**Figure 9:**
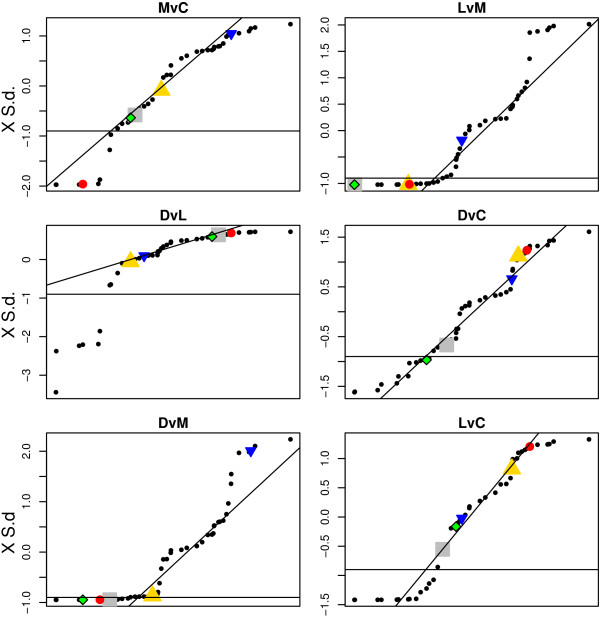
**QQ plots of the standardized p-values versus the standard deviation across comparisons for each protein *set***. For each of the six comparisons of interest a regression line is added which is based on the standardized p-values values between the first and third quartile. To assess which proteins *sets *that were different between comparisons we applied the same relative cut-off value of -0.9 (× S.d.) for all comparisons (black horizontal line). The three *sets *of proteins associated to energy metabolism (glycolysis (G), alcohol metabolism (AM) and tricarboxylic acid cycle (TCA)) were different in parkinsonian animals compared to L-DOPA treated parkinsonian animals (*LvM*). The closely related proteins *sets *for alpha and beta tubulins display different response for the *LvM *comparison.(*Sets *of: G = grey square, AM = green and black diamond, TCA = red solid circle, alpha tubulins = yellow triangle point-up, beta tubulin = blue triangle point down,)

There were differences in the average level of the p-values for the comparisons examined. Therefore it may not be appropriate to use the same cut-off p-value across comparisons. Instead an alternative method using a quantile- quantile -plot (qq-plot) with standardized p-values versus the standard deviations of the p-values for each protein *set *was used (Figure 9). Based on visual inspection of the results for all comparison we selected the same relative cut-off level (-0.9 ×standard deviation, S.d.) across the six comparisons.

### Protein sets

We wanted to explore if the method used herein (DEPPS) can provide insights into the priming effects of L-DOPA in the striatum of a parkinsonian animal model.

Four of the *sets *identified in the *LvM *comparison (Figure 9) were *sets *of proteins that are involved in energy metabolism or tubulin cytoskeleton. We will briefly discuss the possible involvements of these four *sets *of proteins in relation to the priming effects of L-DOPA. The main biological aspects of this study will be published separately (manuscript in preparation).

### Example I: Energy metabolism

**Figure 10:**
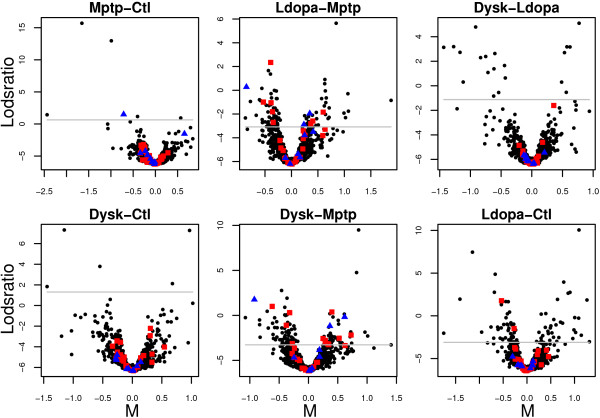
**Volcano plots for proteins involved in energy metabolism**. Proteins involved in G and AM are color coded red (squares) and TCA are color coded blue (triangles). Cut-off levels in the lodsratio corresponding to FDR* equal to 0.30 (red) is plotted for all comparisons. For the *LvM *and *DvM *there is an 'enrichment' of G and AM proteins with higher lodsratios compared to e.g. the *DvL *comparison.

**Figure 12:**
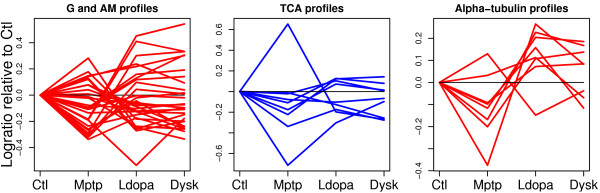
**Profiles for proteins involved in energy metabolism and alpha tubulins**. Profiles showing the effect on proteins involved in energy metabolism and alpha tubulins in parkinsonian animals (Mptp), after one de novo L-DOPA dose (Ldopa) and after long-term L-DOPA treatment resulting in dyskinesia (Dysk). The expression changes are relative the control animals (Ctl). G profiles are proteins involved in glycolysis, AM for proteins involved in alcohol metabolism and TCA profiles are proteins involved in the citric acid cycle.

Three *sets *of proteins involved in energy metabolism (glycolysis (G), tricarboxylic acid cycle (TCA) and alcohol metabolism (AM)) were affected in parkinsonian animals compared to single and long-term L-DOPA treatments (Figure 9, *LvM *and *DvM *and Figure 10). It should be noted that there is a great overlap for proteins classified into the *sets *for glycolysis and alcohol metabolism. For proteins involved in alcohol metabolism, this difference is also maintained after long-term L-DOPA treatment, compared to untreated animals (Figure 9, *DvC*). There is no common expression pattern for these *sets *of proteins, which is not surprising since the energy metabolism comprises many complex pathways (Figure 12).

The changed state of proteins associated with energy metabolism is encouraging since Crossman and co-workers [19] analyzed 2-deoxyglucose accumulation and reported metabolic changes in the basal ganglia in MPTP and L-DOPA treated macaques, with the exception of the striatum which was not studied. Other studies have reported an increase of lactate in the striatum of parkinsonian models, indicating anaerobic glycolysis [20,21]. Metabolic pathways are known to be affected in several neurodegenerative disorders, for a review of recent proteomic findings see [22].

### Example II: Tubulin cytoskeleton

**Figure 11:**
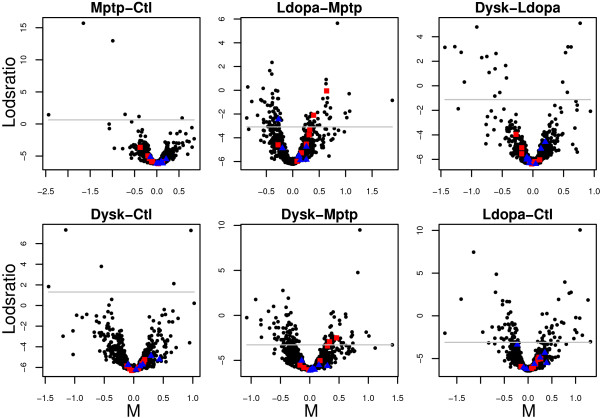
**Volcano plots for proteins involved in tubulin cytoskeleton**. Proteins involved in Alpha and Beta tubulins are color coded red (squares) and blue (triangles). Cut-off levels in the lodsratio corresponding to FDR* equal to 0.30 (red) is plotted for all comparisons.

Proteins that form part of the microtubule cytoskeleton in striatal tissue appear in this study to be affected by single and long-term L-DOPA administration to parkinsonian animals (Figure 9, *LvM *and *DvM *and Figure 11).

The actual difference in protein expression is low, but the protein expression pattern is similar for most proteins in the *set *(Figure 12). The alpha tubulins are expressed/present to a lower level in parkinsonian animals (Mptp) compared to untreated (Ctl), which appear to be reversed after L-DOPA treatment (Ldopa). In contrast to alpha tubulins, beta tubulins do not appear to be affected (Figure 9). The difference in biological response between alpha and beta tubulins suggests different functions.

Dysfunctions of the neuronal cytoskeleton, especially the microtubule system, have been associated with several neurodegenerative diseases such as Alzheimer disease and Amyotrophic Lateral Sclerosis (ALS) [23,24]. Furthermore, MPTP and its metabolites destabilize microtubules [25]. This is interesting since dopaminergic neurons are particularly sensitive to microtubule destabilizing agents [26]. One other possibility is that dopamine regulates changes in the striatal medium spiny neuron (MSN) dendritic trees and spines resulting in changes to the cytoskeleton. Dopamine depletion results in a reduction of spines and synapses on striatal projection neurons [27]. Postmortem samples from Parkinson's patients also show reductions in MSN dendritic spines and dendritic tree size [28]. However, we do not know if the microtubule changes seen in the striatum after L-DOPA treatment in this study are based in dopaminergic fibers from the substantia nigra or in striatal medium spiny neurons and/or interneurons.

## Discussion

### Normalization and spot matching

In the field of cDNA microarrays, spatial and intensity effects arising from printing, hybridization, scanning and other technical factors are known to mask the data obtained from gene expression.

The same applies for protein studies using 2D-DIGE, although the bias arises from different sources. Recent studies by Fodor [9] and Kreil [10] have shown the need for proper normalization and the need for development of better normalization techniques.

Comparing the results by Kreil [10] to ours, their biological example had more dye-bias, however they also included more spots with low spot volume and there is more dye bias in spots with low spot volume. We had a low number of spots per gel with a low spot volume (<40000), on average less than 2%. The differences compared to the study by Kreil may be due to different biological samples, the pH interval for the gel strips, the gel concentration, the settings during scanning or a different setting in the DeCyder software for spot detection.

The fact that we have generally fewer low-volume spots in our data can also be explained by us having manually checked the matching for all spots between the gels in the multi gel interface in the DeCyder software (also known as BVA, Biological Variance Analysis). In our experience the automatic matching needs to be extensively checked, since it is not uncommon with bad matching between spots in different gels, and low volume spots are often surrounded by other low volume spots, making the BVA matching even more unreliable. A large gel set requires a great deal of manual work to minimize the risk of bad matching and it is often that low-volume spots fail to be correctly matched, simply because they can not be found with great confidence between gels.

The methods provided by Fodor [9] and Kreil [10] both make use of the relationship between the dye-bias and intensity but independently of spot location. We think there is a risk of masking the true signals using 2D-DIGE due to spatial bias. The '2D loess+scale' and 'SC-2D+quantile' methods proposed in this report are two new methods that remove both the intensity and spatial bias found in 2D-DIGE data.

In cDNA microarrays the probes are often spotted in a random manner across the microarray, so no spatial bias may be expected. However using 2D-DIGE, spatial bias may to some extent be expected. Certain proteins may be identified in the spot maps as tight rows of spots of proteins originating from the same gene-product (PTMs). They appear in rows in the gels because they have approximately the same mass but different charge.

The spatial bias we found in the raw data and in several normalization methods showed that large areas of spots had higher intensities with either Cy3 or Cy5, irrespective of the treatment group identity of the samples. However, in a case where two very different protein samples are compared on the same gel, then the assumptions made for normalization may not be valid. It can therefore be very helpful to study the spatial plots of un-normalized data, especially if both technical and biological replication is available. See also [29] for further discussions about spatial trends in gel based data.

There are several sources that may cause the spatial bias, such as variations in the quality of the gels, the labeling efficiency of the dyes, incomplete number of proteins transferred from the first to the second dimension and the scanning procedure. It is also known that background subtraction may introduce bias [30]. The procedure for background subtraction using the DeCyder software is to subtract the lowest tenth percentile of the pixel values on the spot border. We have not been able to evaluate the impact of this on the data, since it is not possible to disengage background subtraction using the DeCyder software. See also [31] for a review on sources of variation in gel-based proteomics.

It is unclear from the present study which normalization method gives the most accurate results for each particular protein. However, in most cases the data used to find differentially expressed proteins should not be intensity or spatially biased. The '2D loess+scale' and 'SC-2D+quantile' methods proposed in this study are the only methods that sufficiently satisfy these two criteria.

### Loess smoothing and possible software improvements

When using loess smoothing [32], the smoothing factor has to be set by the user. This factor (also known as span) indicates the percentage of spots to be used in the estimation of each point of the loess curve.

For the master gel (gel 4) a smoothing factor between 0.05 and 0.1 satisfyingly removed both the intensity and spatial bias. Using the same smoothing factor on a gel with only half as many spots, the estimation of each point would be performed on relatively fewer spots and there may be a risk of over fitting. To minimize the risk of over fitting a smoothing factor proportional to the number of spots is appropriate. In this case a factor of e.g. 100/[number of spots], gives a higher span for gels with less spots. This may seem as a relative low smoothing factor, but the spatial bias found in our gels are relative local. A smoothing factor that is too high would result in a more global intensity biased normalization rather than local bias normalization.

For ideal 2D-DIGE spatial (and global) normalization all spots identified in each gel should be used for normalization, instead of only using spots also found in the master image. This would probably improve the local bias normalization slightly and therefore give a better final result. Using version 5.02 of the DeCyder software this is not possible because the merging of two spots in the multi-gel comparison interface, BVA, results in no update of the original spot map. Consequently the normalized value can not be extracted. If no spots in any of the gels have been merged it is possible to use all spots, but in our experience this is seldom the case.

### The pool channel and parameter estimation

The DeCyder software manual, as well as Fodor *et al *[9], recommends the pool channel (Cy2) to be used in the statistical analysis for all types of gel experiments.

After normalization ('DeCyder pool' and 'Fodor'), the pool channel intensity will generally not cancel out when the logratios are combined, but there will be an error term connected to each of log_2_*Cy5*/*Cy2 *and log_2_*Cy3*/*Cy2*. Karp *et al *[33] even suggests that the Cy2 channel gives more noise than each of Cy3 and Cy5, in which case the noise added in each step of log_2_*Cy5*/*Cy2 *and log_2_*Cy3*/*Cy2 *would be higher than the single noise term in the direct measurement log_2_*Cy5*/*Cy3*.

The linear model is then based on the logratios log_2_*Cy5*/*Cy2 *and log_2_*Cy3*/*Cy2 *rather than directly on the logratios between the red and the green intensities (log_2_*Cy5*/*Cy3*) only or on the log intensities log_2_*Cy2*, log_2_*Cy3 *and log_2_*Cy5*. According to the DeCyder software manual, as well as Fodor *et al *[9], these logratios may be considered as independent measurements.

We want to stress that if a log difference between only two samples on the same gel is to be measured, the standard error of the measurement will be larger if the difference is estimated via a third (pool) sample than if it is estimated directly between the samples of interest. Additionally the logratios log_2_*Cy5*/*Cy2 *and log_2_*Cy3*/*Cy2 *can not be considered as independent measurements, since they originally come from the same gel.

As a consequence, in a case where only two treatments are compared with replicate gels, we advocate the use of the pool channel (Cy2) for mapping between gels and the use of normalized *M*-values (log_2_*Cy5*/*Cy3*) in the linear model.

In a case where more than two treatments or states will be compared the situation may be different, then the analysis may improve on including the pool channel in the linear model. We recommend the data first be normalized using 'SC-2D+quantile' and then the three log_2 _intensity estimates for each spot on a gel should be treated as correlated observations. As a consequence the three dyes should be treated as fixed effects and the gels should be treated as blocks by a random effect in the linear model.

Experiments should be carefully designed so that the comparisons of particular interest are made within gels rather than between gels. The labeling using different dyes (especially Cy5 and Cy3 if Cy2 is used for the pool samples) of the experimental groups should also be balanced. Since our experiment was originally designed following only the manual of the DeCyder software (which then assumed no dye bias), little attention were given to the possibility of systematic bias within the experiment and the balance of the dyes for each experimental group in the design.

See also microarray statistics theory on direct versus indirect experimental designs in e.g. [34,35]

### Protein identification and predefined sets of proteins

Traditionally, studies of gene expression have drawn biological conclusions from lists of differentially expressed genes. The development and use of a more pathway-oriented approach in gene studies, such as GSEA[14], have recently become more popular.

Most 2D-DIGE studies have focused on identifying individual spots (proteins) that are differentially expressed between two states. Using this approach it is hard to make conclusive remarks about effects in biological processes and pathways.

We have used a pathway orientated approach (DEPPS) in this 2D-DIGE study. The ideal situation would have been to have all spots in the gels identified, but this is quite difficult, especially for the low volume spots. There are several reasons for our relative low number of identified spots with low spot volume. The main reason is that the spots were manually picked from preparative Coomassie stained gels (see Methods: Spot picking and digestion). The Coomassie staining is less sensitive compared to the CyDye staining. The lesser sensitivity can not be completely compensated for by loading more proteins on preparative gels. Consequently spots with low spot-volumes in the 2D-DIGE gels could not be found in the preparative gels, at least not with a great certainty that they were the same spots.

Another reason is that the spots were manually extracted from the preparative gels in a random and unbiased way *before *any statistical analyses were made. Spots that had been picked for identification were afterwards matched to the 2D-DIGE gels. It may have been possible to first find the spots in the 2D-DIGE gels, then try to match these low spot-volume proteins (between 2D-DIGE and preparative gels) and then pool spots from several preparative gels to get enough of protein for protein identification. This was not done due to the extensive workload needed.

Although some interesting low-volume spots may have been missed for protein identification, we think that looking at differences between groups in *sets *of proteins has revealed confirmatory and new intriguing results in an animal model of Parkinson's disease. This is in spite of the quantitatively small differences between the treatments. These results would probably not have been found when looking only at single differentially expressed proteins.

### The false discovery rate (FDR) and significance levels

The issues of significance levels and multiple testing are often revisited in the literature of gene expression, with varying conclusions drawn. In 2D-DIGE gels, where location and content of spots are unknown before the experiment is carried out, the complexity is further increased compared to gene arrays. A spot/protein originating from the same gene-product may be represented in several positions in the gels due to PTMs and artifact spots may also be present. Therefore the spots, and hence the tests, may not be considered independent. As a consequence we give little confidence in the determined significance levels or false discovery rates.

To address this problem we derived potential lodsratios cut-off for each comparison through a permutation test. This test can be used as guidance for deriving cut-off levels, especially for studies where only a limited number of spots will be identified. However, we still prefer to see the observed single spot lodsratios solely as rankings of the evidence for changes in protein activity. To detect changes in biological processes for known and well characterized proteins we recommend using methods such as DEPPS which makes use of the whole ranking list.

## Conclusion

This study demonstrates that there may be substantial intensity and spatial bias in 2D-DIGE data. The '2D loess+scale' and 'SC-2D+quantile' are the only normalization methods evaluated in this study that sufficiently remove both the intensity and spatial bias.

For direct comparison between two treatments or states we recommend the use of normalized *M*-values and that the commonly used pool channel (Cy2) should be used for mapping between gels only.

When more than two treatments or states will be compared the pool channel (Cy2) may be included in the linear model but this depends on the original study design and comparisons of interest. The three log_2 _intensities of the dyes should then be treated as correlated single channel fixed effects and the replicate gels should be treated as a random factor in the linear model.

Different methods correcting the observed significance levels are used for 2D-DIGE data. We like to stress that the gel spots, and hence the tests, can not be considered as independent measurements. As a consequence we recommend the p-values in significance tests in 2D-DIGE data to be used as rankings only and that looking at *sets *of proteins instead of individual proteins generates more accurate and biologically informative results.

Using the DEPPS method which is based on *sets *of proteins; we found that proteins in the striatum that are involved in energy metabolism and tubulin cytoskeleton appear to be affected by the administration of L-DOPA in the golden-standard animal model of Parkinson's disease.

## Methods

### Animal treatment

All animal studies were carried out as described in [36] in accordance with European Communities Council Directive of 24 November 1986 (86/609/EEC) for the care of laboratory animals. A total of 27 female monkeys (*Macaca fascicularis*, SAH, Beijing, China; average age = 4.4 years (between 4–7 years); mean weight = 2.9 kg (2.4–3.4 kg)) were used and rendered parkinsonian and dyskinetic according to published methods [19,37-41]. Six animals were kept as a control group and the remaining 21 animals were injected with MPTP hydrochloride until bilateral parkinsonian symptoms of comparable severity were stabilized (mean cumulative dose of 2.44 mg/kg). Ten animals were dosed with L-DOPA (Modopar^®^, Roche, L-DOPA/carbidopa, ratio 4:1) twice daily (approximately for 4.5 months). The L-DOPA dose was tailored to produce a full reversal of the parkinsonian condition (20–60 mg). All ten animals exhibited L-DOPA induced dyskinesia and received their final tailored dose of L-DOPA one hour before death. Eleven animals were kept without L-DOPA administration for approximately 4.5 months. Six of these animals received a single dose of L-DOPA (50 mg) one hour before death. All animals were killed with a sodium pentobarbital overdose (150 mg/kg, i.v.). Dissection of different brain regions were performed on ice with the brain immersed in cold saline (0.9%) in less than 15 min. The striatum (combining caudate nucleus, putamen and nucleus accumbens, across the rostrocaudal extent of the structure) was dissected from each hemisphere, immediately frozen at -45°C in isopentane and then stored at -80°C.

### Sample preparation

Each frozen striatum was taken directly from the freezer, put in an eppendorf-tube, and rapidly homogenized in a 4:1 (v/w) ratio of lysis buffer containing 8 M urea, 4% 3-[(3-Cholamidopropyl)Dimethyl-Ammonio]-1-Propanesulfonate (CHAPS), 70 mM dithiothreitol (DTT), 5% immobilized pH gradient (IPG) buffer pH 3–10, using a sonicator. The sonication was performed on ice (to avoid carbamylation of the proteins) in pulses for 10 seconds (Fisher Bioblock scientific), followed by ultracentrifugation for 1 hour at 100 000 × g (Beckman Optima, Beckman). Supernatants were collected and cleaned from lipids and nucleic acids using the 2D Clean-up Kit (GE Healthcare, Uppsala, Sweden), according to the manufacturer's instructions. The total protein concentration of each sample was determined using the 2D Quant Kit (GE Healthcare) in accordance with the manufacturer's protocol. The whole procedure was performed on ice whenever possible to minimize protease activity.

### Design

This study consists of 14 2D-DIGE gels. Each gel contains two striatum samples from different animals. The samples have either been labeled with the red (Cy5) or the green (Cy3) fluorescent dye. Additionally, all gels contain a third sample which is a pool of all the samples in the experiment. Equal amounts of proteins from each of the individual sample were used for the pool. This pool sample is labeled with the yellow (Cy2) fluorescent dye and plays an important role in the matching of spots between gels. It is also included in some of the normalization methods evaluated in this paper and in some of the statistical methods for parameter estimation.

The experimental design (the distribution of the samples between gels) is tabulated in Table 1. All the Cy5 and Cy3 labeled samples are biological replicates but one; the Cy5 labeled sample used on gel 14 is a technical replicate of the control animal, which is also represented on gel 4. The technical replicate was not treated differently from the biological replicates, since we believe the gains would be marginal in the otherwise so complex system. The gels were run six at a time and the final two last.

**Table 1:** 2D-DIGE experimental design

Gel	Cy3	Cy5
1	Ctl1	Ldopa2
2	Ctl2	Dysk5
3	Ctl3	Ctl4
4	Ctl5	Mptp1
5	Ctl6	Mptp3
6	Mptp2	Ldopa4
7	Mptp4	Dysk10
8	Mptp5	Dysk1
9	Ldopa1	Dysk6
10	Ldopa3	Dysk9
11	Ldopa5	Dysk2
12	Ldopa6	Dysk7
13	Dysk3	Dysk8
14	Dysk4	Ctl5

Each gel had three samples. Two corresponding to a sample of interest (labeled with Cy3 or Cy5) and a pooled standard which is a mix of all samples used in the study (labeled with Cy2). The samples were from four different groups of animals: untreated animals (Ctl), those treated with Mptp (Mptp), those treated with Mptp plus one acute dose of L-DOPA (Ldopa) and finally those treated with Mptp and long-term L-DOPA treatment (Dysk).

### Gel preparation

For 2D-DIGE we labeled 50 μg each of control, treated, and pooled protein sample with cyanine dye Cy5 or Cy3 and Cy2, respectively, according to the manufacturer's descriptions for CyDye DIGE Fluor minimal dyes (GE Healthcare). The pooled sample was a mixture of equal amounts of protein from all samples in the experiment. Before the first-dimension isoelectric focusing (IEF), a 50-μg aliquot from each of the three labeling mixes (see above) was combined with DeStreak rehydration buffer and 0.5% (v/v) Pharmalytes (GE Healthcare) that covered the pH interval (pH 3–11 NL) of the IPG strips, to give a final volume of 450 μL.

Gel rehydration of the 24-cm IPG strips (GE Healthcare) with the 450-μL rehydration buffer (including the protein sample), was performed at room temperature in the dark for 12 hr according to the manufacturer's instructions. IEF was run on an IPGPhor (GE Healthcare) at 500 V for 1 hr, at 1 kV for 1 hr, and at 8 kV until a total of 64 kVh was reached. After IEF, the strips were equilibrated for 2 × 15 min by gentle shaking in a buffer containing 50 mM Tris-HCl (pH 6.8), 6 M urea, and 2% sodium dodecyl sulfate (SDS), supplemented with 2% DTT in the first equilibration step and 2.5% iodoacetamide in the second. For the second dimension SDS-polyacrylamide gel electrophoresis (SDS-PAGE), the equilibrated strips were put on top of large format 12.5% polyacrylamide gels and were run using an Ettan DALTsix large-format vertical system (GE Healthcare). The gels were run at 5 W for 45 min before increasing to 11 W per gel until the bromophenol blue dye front had reached the bottom of the gel. The temperature was kept constant at 27°C. The gels were then subjected to image analysis.

### Scanning and image analysis

All gels were scanned using a Typhoon 9400 (GE Healthcare) at 100 μm resolution. The images were analyzed using the DeCyder software suite (GE Healthcare, version 5.02). All 14 × 3 images were loaded into the DeCyder Batch processor, and the program was set to find 3000 spots in each image then filter away artifacts and finally to do a primary matching between all the different gel images. The resulting files were then loaded into the BVA module for further image analysis.

All spots were manually compared between the different gels to minimize false spot matching. The gel with the largest number of spots identified was used as the master gel. When needed, spots were merged to better match against the spots in the master gel. Volume data, DeCyder normalized data, and coordinate data for each spot was exported using the DeCyder XML toolbox.

### Spot picking and digestion

Preparative gels containing 500 μg proteins were stained with Colloidal Coomassie Brilliant Blue G (Acros Organics, Geel, Belgium) and matched to the fluorescent 2D-DIGE images. Spots were manually extracted from a total of six preparative gels (n = 6). As many spots as possible recognizable in both 2D-DIGE and preparative gels were extracted. Gel pieces (spots) were washed twice (0.2 M NH_4_HCO_3_/50% ACN) with 30 min incubation at 30°C. Subsequently, the gel pieces were dried by SpeedVac concentration (Concentrator 5301, Eppendorf) and trypsinated (0.4 μg trypsin/gel piece (Modified Sequence Grade Trypsin, Promega)) followed by overnight incubation in 30°C. Trypsin activity was stopped by addition of trifluoroacetic acid (TFA) to an final concentration of 1%. Peptides were extracted with 60% ACN/0.1% TFA followed by complete drying by SpeedVac to remove organic solvents. The peptides were then re-suspended in 15 μl 0.25% Acetic acid (HAc).

### Protein identification

The tryptic digests from each spot were dissolved in 10 μl 0.25% (v/v) acetic acid. Five μl was desalted on a Nano-Precolumn (LC Packings, Amsterdam, the Netherlands) using Ettan MDLC (GE Healthcare). The digest was then separated by a 20 minute gradient from 3 to 80% acetonitrile in 0.25% acetic acid on a 15 cm, 75 μm inner diameter C18 capillary column (LC Packings, Amsterdam, the Netherlands). At a flow rate of approximately 150 nl/min the peptides were electro sprayed into a linear ion trap mass spectrometer (LTQ, Thermo Electron, San Jose, CA, USA). The spray voltage was 1.8 kV, ES source capillary temperature was 160°C, and 35 units of collision energy were used to obtain peptide fragmentation. One zoom scan spectrum and one tandem mass spectrometry (MS/MS) scan spectrum were collected in a data dependent acquisition manner following each full-scan mass spectrum. The dynamic exclusion feature enabled sequence information of as many co-detected peptides as possible.

The information from the electro spray ionization MS and MS/MS spectra were correlated to protein and translated DNA sequence data in the UniProt database using Mascot. The non-redundant sub database of *Homo sapiens *was used with the parameters as follows: partial oxidation of methionine (+16 Da), and cysteine alkylation (+57 Da), peptide mass tolerance of 1.5 Da and fragment ion mass tolerance of 0.8 Da. Trypsin was specified as the digesting enzyme with a maximum of one missed cleavage. The criteria for positive identification of a protein were two or more peptides with each a Mascot score of 33 or higher from the same protein. The full list of all identified proteins and their peptide Mascot scores will be published separately (manuscript in preparation).

### Protein classification

All identified proteins were manually categorized using the information, when available, provided by the Gene Ontology project [42] and related information from the scientific literature. The classifications of the proteins were based on molecular function, their involvement in biological processes and/or cellular localization. The classifications were solely based on similarities in function and biological processes of all the proteins as seen by the authors. Literature references used in classifications that are not solely based on Gene Ontology (GO) will be available in the biological interpretation of the data in this study (manuscript in preparation). Based on the information available on the identified proteins, 47 classes were identified. A single protein may have been classified into several different *sets*.

### Methods for normalization

In the field of two channel cDNA microarrays, normalization methods seek to ensure that systematic variation, such as dye effect, are removed while biological variation is retained.

After normalization the fluorescent dye intensities are expected to be balanced, that is show equal amount of signal corresponding to equal gene expression levels. This assumption is valid in cDNA microarrays only if a small proportion of the spots (genes) are different between treatments, and if spots are printed in a random order on the microarray slide.

For the normalization of data from 2D-DIGE we assume that only a small proportion of the spots/proteins are different between treatments and that they do not appear in a systematic spatial pattern (violations to this assumption may in some cases be expected, though. See also Discussion; Normalization). In this study we have evaluated eight different normalization methods, whereas four of them have not previously been used on 2D-DIGE data.

The DeCyder software (GE Healthcare) provides two methods for data normalization. We call them 'DeCyder no pool' and 'DeCyder pool' respectively. The 'DeCyder no pool' method is recommended only when there is no pool channel included in the experiment. The method consists of channel specific shifts in log intensities (log volumes), so that the distribution of log intensities gets centered on zero for each dye channel. The 'DeCyder pool' performs equivalent shifts on each of the two series of logratios log *Cy5*/*Cy2 *and log *Cy3*/*Cy2 *from each gel.

Two different normalization strategies for 2D-DIGE data have recently been suggested in Kreil *et al *[10] and Fodor *et al *[9], we will name the methods 'Kreil' and 'Fodor' respectively. Their strategies were first developed for the normalization of two channel cDNA expression data.

'Kreil' do not include the pool channel. Variance stabilization normalization (vsn) [4] is applied to each red (*Cy5*) and green (*Cy3*) channel separately, followed by a median shift [8] of the gel specific logratios log *Cy5*/*Cy3 *and finally standardization of the logratio distributions of all the gels to an equal scale by a robust Z-score.

'Fodor' first applies 'DeCyder pool' normalization but then adjusts the gel specific 'standardized log abundances', log *Cy5*/*Cy2 *and log *Cy3*/*Cy2*, for within gel intensity dependence with a loess smoother [32], and scales the resulting gel specific quantities (log *Cy5*/*Cy2*)'-(log *Cy3*/*Cy2*)' to equal scales for the middle 50 percent of these quantities for each gel [8].

We have also tested four normalization methods that have not been used on 2D-DIGE data before. We call the first two methods 'loess + scale' and '2D loess + scale'. Yang *et al *[8] introduced the robust scatter plot smoother loess [32], to adjust for intensity bias in two channel cDNA microarray expression data. In two-channel spatial loess normalization a loess smoother function is estimated from the differences (*M*) between the (log_2_) dye intensities, log_2_*Cy5*-log_2_*Cy3*. The estimated loess function is then subtracted from the differences (*M*), and the separate log_2 _intensities, log_2_*Cy5 *and log_2_*Cy3 *can be recovered. In effect, the method comes down to shifting both of the two sets of log-intensities (log_2_*Cy5*) and (log_2_*Cy3*) to their average (log_2_*Cy5*+log_2_*Cy3*)/2, except noise and true differential expression are still present. The scaling technique by Yang *et al *[8]which adjusts the scales of the logratio distributions to equal levels between the microarray slides, is seldom needed in microarray analysis, but we see a more general need for such scaling between 2D-DIGE gels, therefore we also perform scaling between gels. For the 'loess + scale' normalization, we used the function normalizeWithinArrays with default settings and normalizeBetweenArrays using 'scale' found in the software R [18] Limma package [7].

The '2D loess + scale' is a spatial location normalization method. The loess smoother function is now estimated from the differences (*M*) between the (log_2_) dye intensities, log_2_*Cy5*-log_2_*Cy3*, as a 2-dimensional function of the spot coordinates on the protein gel. The estimated loess function is then subtracted from the differences (*M*). The method eliminates the phenomenon of one dye showing generally higher values than the other across regions of the gel. For the loess smoother estimation we make use of all of the spots that are also found in the master gel (see also Discussion: Possible software improvements). For the spatial location normalization we used the function ma2D in the marray R package [3]. The coordinates for each spot was extracted from the DeCyder software. The smoothing factor was calculated as the ratio 100/[number of spots also found in the master image] for each gel. The data was finally scaled between the gels, as described above.

The final two methods make use of the raw intensities for all three dyes. The first method 'SC-quantile' (for Single Channel quantile normalization) was developed for single-channel Affymetrix data [43] and it ensures that the intensities for the three dyes have the same empirical distribution across gels. The second method 'SC-2D+quantile', first applies spatial location normalization and then single channel quantile normalization.

To perform spatial normalization for the three dyes, we extended the two channel loess methods described above to suit three (or more) channels. We performed spatial loess normalization with three dyes such that all the three sets of log_2_-intensites from a spot (log_2_*Cy5*, log_2_*Cy3 *and log_2_*Cy2*) were shifted to the average intensities (log_2_*Cy5*+ log_2_*Cy3*+ log_2_*Cy2*)/3, except noise and true differential expression were kept. In practice this was done using the ma2D function in the marray R package [3] three times. Instead of the two sets of log_2_-intensites log_2_*R *and log_2_*G*, we supplied one set of log_2_-intensities (log_2_*Cy5 *or log_2_*Cy3 *or log_2_*Cy2*) as well as the set of averages (log_2_*Cy5*+ log_2_*Cy3*+ log_2_*Cy2*)/3 to the function ma2D, so that the log_2_-intensites would be subtracted by the averages, and the loess function fit to and subtracted from the resulting differences. The normalized log_2_-intensites were then derived by adding the averages (log_2_*Cy5*+ log_2_*Cy3*+ log_2_*Cy2*)/3 again to the output of the ma2D. The same idea can be used to generalize loess normalization of intensity bias to settings with more than three channels. After loess normalization the single channel data was also quantile normalized as described above.

We have used diagnostic plots to examine the raw data and the ability of the different normalization methods to remove intensity and/or spatial bias. We will normally calculate *M*-values as log_2_*Cy5*/*Cy3 *but for method 'Fodor' and 'DeCyder pool' which includes the pool channel (*Cy2*) *M *will be calculated as log_2_(*Cy5*/*Cy2*)/(*Cy3*/*Cy2*). We will consequently define *m*-values as log_2_(*Cy5*/*Cy2*) or log_2_(*Cy3*/*Cy2*). The average log_2 _intensity (*A*) is calculated as(log_2_*Cy5*+log_2_*Cy3*)/2.

To compare different methods we used box plots of *M *and *m*-values, scatter plots of *M *versus *A*-values (MA plots) and color coded spatial gel plots that depicts *M*-values and the coordinates for each spot given in the DeCyder software (2D-M plots).

For the single channel analysis we calculated three logratios for each spot:

*MCy5 *= log_2_*Cy5*/*I*, *MCy3 *= log_2_*Cy3*/*I *and *MCy2 *= log_2_*Cy2*/*I*, where

*I *= (log_2_*Cy5*+ log_2_*Cy3*+ log_2_*Cy2*)/3.

To compare methods for single channel analysis we used box plots of all three log_2 _intensities, scatter plots of *MCy5*, *MCy3 *and *MCy2 *versus *I*-values (3Dyes-I plots) and color coded spatial gel plots that depicts *MCy5*, *MCy3 *and *MCy2*-values and the coordinates for each spot (2D-Intensity plots).

### Parameter estimation

Our interest was in comparing protein expression across four distinct groups of target samples; striatum in the basal ganglia from untreated animals (Ctl), from those treated with MPTP (Mptp), striatum from those treated with MPTP plus one acute dose of L-DOPA (Ldopa) and striatum from those treated with MPTP and long-term L-DOPA treatment (Dysk).

We were interested in six different comparisons; those between Mptp and Ctl, Ldopa and Mptp, Dysk and Ldopa, Dysk and Ctl, Dysk and Mptp and Ldopa and Ctl. We called the corresponding parameters *MvC*, *LvM*, *DvL*, *DvC*, *DvM *and *LvC *respectively, so that e.g. *MvC*_*p *_represented the expected difference in expression levels between the Mptp and Ctl treatment groups for protein *p *on a log_2 _scale.

For each spot/protein *p*, we based our statistical analysis on the *log*_2_-intensites (log_2_*Cy5*_*p1*_,..., log_2_*Cy5*_*pJ *_, log_2_*Cy3*_*p1*_,..., log_2_*Cy3*_*pJ *_, log_2_*Cy2*_*p1*_,..., log_2_*Cy2*_*pJ*_) where *p *= 1,..., *P *is a spot/protein index and *j *= 1,..., *J *is a gel index. The gels were treated as blocks in the linear model, so that effects were estimated within gels where possible. Hence a mixed model was set up with gel as random factor (*β*_*j*_, *j *= 1,...,14), dye as fixed factor (*F*_*k*_, *k *= 1,...,3) to account for differences between the channels Cy5, Cy3 and Cy2 and fixed effects *T*_1_,...,*T*_4 _for the four treatment states Ctl, Mptp, Ldopa and Dysk. Let, for example, *Z*_*pjkl *_denote the log_2_-intensity for the spot/protein *p *on gel *j*, channel *k*, which happens to reflect an expression level under treatment *l*. Then we let

*Z*_*pjkl *_= *β*_*j *_+ *F*_*k *_+ *T*_*l *_+ *ε*_*pjk*_,

where *ε*_*pjk *_is an error term which we assume follows a normal distribution *ε*_*pjk*_~ iid N(0, *σ*^2^_*εp*_). We also assume that *β*_*j *_follows a normal distribution, but with a different variance, *β*_j_~ iid N(0, *σ*^2^_β*p*_). The pool channel samples, which originally were created by taking equal amounts of proteins from each sample and mixing, were consequently assumed to contain 6/27 Ctl, 5/27 Mptp, 6/27 Ldopa and 10/27 Dysk.

After the data had been normalized using 'SC-2D+quantile', the six comparisons (or effects) of interest were estimated by the least squares method using the lmFit Limma function [6]. The correlation between the spots of the three dyes within each gel was first estimated using the function dupcor [44] in Limma. To test for differences between the expression levels in the treatment groups we calculated lodsratios (*B*_*pl*_) for each protein *p *and effect *l *for the effects *MvC*, *LvM*, *DvL*, *DvC*, *DvM *and *LvC *using eBayes in Limma, (see [5,6]).

The eBayes function is a further development on the method proposed by [5]. This empirical Bayes lodsratio or equivalently smoothed (penalized) t-statistic [6] was first developed for gene expression data and it is based on an empirical Bayes estimate of the standard error. An increasing differential expression (increasing *M*) increases the lodsratio (for log-odds ratio) all the more if the variance is small. However if *M *is small too, a factor in the model ensures that the lodsratio cannot blow up due to small variances. Using only the average *M *values as statistic for differential expression some spots will be driven by outliers and using the ordinary t-statistic others will be strongly influenced by small standard error estimates. Empirical Bayes statistics can rule out both of these categories of spots (genes or proteins).

It is important to include as many spots as possible, since they add information about the distribution of (particularly noise) expression level differences, which is needed in the calculation of the lodsratios. However, we still recommend the spots between gels to be carefully mapped as this minimizes the risk of including wrongly mapped spots and non-sense data. The mapping of spots can be very time-consuming, but in our experience this also lowers the number of hypothesis tests that later have to be made. We included spots for which we had enough observations to estimate all possible comparisons between treatment groups, and for which the degrees of freedom in the linear model was at least eleven.

The full spot list was used to calculate lodsratios although not all spots had been identified. We assume that small spots behave vaguely like spots of proteins with low expression levels. Although a crude assumption it is probably a good first approximation. For each comparison a volcano plot was made, which shows the calculated lodsratios versus the parameter estimate, i.e. the magnitude of the difference.

For the parameter estimation using 'DeCyder pool' normalized data, the blocking effect (*β*_*j*_) was omitted and only the dye effects for Cy5 and Cy3 were included.

### Defining cut-off levels

The goal of most 2D-DIGE studies is to find single regulated proteins between two different conditions, by testing for significant variation between the means or medians. We and others have then used methods for correcting the significance levels, because numerous tests are performed simultaneously [9,13]. However the issue of multiple testing in 2D-DIGE, compared to microarrays, is more complex to address. A protein originating from the same gene product but with different modifications (PTMs) may be represented in several positions in the gels. Merging these spots into a single abundance would possible cause a misinterpretation of the effect on protein level. Since not all spots in the gels have been identified there is also a risk of including data from artifact spots. In summary it is probably not valid to assume the spots, and hence the tests to be independent.

To address this problem we derived potential lodsratio cut-offs for each comparison through the permutation test described next.

A set of 10 000 non-sense datasets were produced by permuting the gel numbers in the real dataset. We then followed the outline described earlier where spots were excluded if there were not enough observations (see Methods; Parameter estimation), and derived the effect estimates and the lodsratios for each dataset. For predefined potential lodsratio cut-off levels in each contrast we registered the number of spots observed above the cut-off in the non-sense datasets.

To adjust for differences in size between nonsense datasets we multiplied these numbers by the number in the real dataset (1211) and divided by the number of spots in respective non-sense dataset. The resulting, adjusted numbers of spots above each specific cut-off was then averaged (to n*, say) over the 10 000 non-sense datasets. The average was compared to the observed number of spots above the cut-off in real data, say *n*. An estimate of the false discovery rate connected to the cut-off was derived as FDR* = n*/*n*.

For each contrast we calculated the lodsratio cut-off levels for the FDR* of 0.30, 0.50 and the minimum observed FDR*.

### Differential expression in predefined sets of proteins

Single differentially expressed proteins and their interpretation may be interesting as such, but studying each protein separately may be difficult and ineffective in many cases. Instead we were primarily interested in the expression changes of predefined *sets *of proteins and their interpretation.

In a paper on genome-wide expression by Subramanian *et al *[14] a method is presented to determine whether members of a predefined gene *set S *tend to show similar degrees of differential expression. The authors developed a non-parametric location test statistic, which is evaluated through a permutation scheme where gene labels are permuted. They named their method Gene Set Enrichment Analysis (GSEA).

Tian *et al *[15] point out that GSEA only tests the genes for similar behavior, whether they show differential expression, non-differential expression or only vague but similar results. Tian *et al *[15] divide the task into two null hypotheses:

Q_1_: The genes in *S *show the same pattern of association with the phenotype as the rest of the genes.

Q_2_: The gene *set *does not contain any genes whose expression levels are associated with the phenotype.

They suggest a test statistic which is the single gene test statistics averaged over the gene set, and test Q_1 _by permuting gene labels (which correspond to our protein labels) and Q_2 _by permuting array labels (corresponding to gel labels).

Our interest is in groups of proteins for which any (possibly few) proteins show association with the phenotype of interest. The proteins in these *sets *do not necessarily have to behave similarly, which would be tested by Q_1_. We therefore test the Q_2 _hypothesis only.

Below we modify the method further and adapt it to protein data. We call our method DEPPS (Differential Expression in Predefined Proteins *Sets*).

For a protein set *S*, we calculate the test statistic

B¯S=1|S|∑p∈SBpi
 MathType@MTEF@5@5@+=feaafiart1ev1aaatCvAUfKttLearuWrP9MDH5MBPbIqV92AaeXatLxBI9gBaebbnrfifHhDYfgasaacH8akY=wiFfYdH8Gipec8Eeeu0xXdbba9frFj0=OqFfea0dXdd9vqai=hGuQ8kuc9pgc9s8qqaq=dirpe0xb9q8qiLsFr0=vr0=vr0dc8meaabaqaciaacaGaaeqabaqabeGadaaakeaadaqdaaqaaiabdkeacbaadaWgaaWcbaGaem4uamfabeaakiabg2da9maalaaabaGaeGymaedabaWaaqWaaeaacqWGtbWuaiaawEa7caGLiWoaaaWaaabuaeaacqWGcbGqdaWgaaWcbaGaemiCaaNaemyAaKgabeaaaeaacqWGWbaCcqGHiiIZcqWGtbWuaeqaniabggHiLdaaaa@3FB7@

for each comparison *i *of interest, where *B*_*pi *_is the lodsratio score for protein *p *and comparison *i*, and |*S*| is the number of proteins in *S*. We compared B¯S
 MathType@MTEF@5@5@+=feaafiart1ev1aaatCvAUfKttLearuWrP9MDH5MBPbIqV92AaeXatLxBI9gBaebbnrfifHhDYfgasaacH8akY=wiFfYdH8Gipec8Eeeu0xXdbba9frFj0=OqFfea0dXdd9vqai=hGuQ8kuc9pgc9s8qqaq=dirpe0xb9q8qiLsFr0=vr0=vr0dc8meaabaqaciaacaGaaeqabaqabeGadaaakeaadaqdaaqaaiabdkeacbaadaWgaaWcbaGaem4uamfabeaaaaa@2F25@ to 10000 equivalent non-sense statistics B¯S*1,...,B¯S*10000
 MathType@MTEF@5@5@+=feaafiart1ev1aaatCvAUfKttLearuWrP9MDH5MBPbIqV92AaeXatLxBI9gBaebbnrfifHhDYfgasaacH8akY=wiFfYdH8Gipec8Eeeu0xXdbba9frFj0=OqFfea0dXdd9vqai=hGuQ8kuc9pgc9s8qqaq=dirpe0xb9q8qiLsFr0=vr0=vr0dc8meaabaqaciaacaGaaeqabaqabeGadaaakeaadaqdaaqaaiabdkeacbaadaqhaaWcbaGaem4uamfabaGaeiOkaOIaeGymaedaaOGaeiilaWIaeiOla4IaeiOla4IaeiOla4IaeiilaWYaa0aaaeaacqWGcbGqaaWaa0baaSqaaiabdofatbqaaiabcQcaQiabigdaXiabicdaWiabicdaWiabicdaWiabicdaWaaaaaa@3D66@ which we got by permuting gel numbers n in the dataset exactly as in the section above (Methods, Defining cut off levels). The full lodsratio ranking list was used when calculating each lodsratio *B*_*pi*_.

A simple significance level (p-value) for each protein *set *was calculated as

∑j=110000I{B¯S*j>B¯S}10000,
 MathType@MTEF@5@5@+=feaafiart1ev1aaatCvAUfKttLearuWrP9MDH5MBPbIqV92AaeXatLxBI9gBaebbnrfifHhDYfgasaacH8akY=wiFfYdH8Gipec8Eeeu0xXdbba9frFj0=OqFfea0dXdd9vqai=hGuQ8kuc9pgc9s8qqaq=dirpe0xb9q8qiLsFr0=vr0=vr0dc8meaabaqaciaacaGaaeqabaqabeGadaaakeaadaWcaaqaamaaqahabaGaemysaK0aaSbaaSqaamaacmaabaWaa0aaaeaacqWGcbGqaaWaa0baaWqaaiabdofatbqaaiabcQcaQiabdQgaQbaaliabg6da+maanaaabaGaemOqaieaamaaBaaameaacqWGtbWuaeqaaaWccaGL7bGaayzFaaaabeaaaeaacqWGQbGAcqGH9aqpcqaIXaqmaeaacqaIXaqmcqaIWaamcqaIWaamcqaIWaamcqaIWaama0GaeyyeIuoaaOqaaiabigdaXiabicdaWiabicdaWiabicdaWiabicdaWaaacqGGSaalaaa@4844@

where *I*_{•} _is equal to 1 if the argument is true and 0 otherwise.

It is difficult to define cut-offs for the p-values (see Discussion; The false discovery rate (FDR) and significance levels). We propose to standardize the p-values for each comparison and plot them in quantile-quantile (qq)-plots versus the standard deviations of the set specific p-values across comparisons.

Let *i *= 1,...,*b *be an index over the comparisons and *j *= 1,...,*n *be an index over the n = 47 protein *sets*. For each p-value *p*_*ij *_we computed standardized p-values as

p'ij=pij−mean(pi1,...,pin)sd(p1j,...,pbj).
 MathType@MTEF@5@5@+=feaafiart1ev1aaatCvAUfKttLearuWrP9MDH5MBPbIqV92AaeXatLxBI9gBaebbnrfifHhDYfgasaacH8akY=wiFfYdH8Gipec8Eeeu0xXdbba9frFj0=OqFfea0dXdd9vqai=hGuQ8kuc9pgc9s8qqaq=dirpe0xb9q8qiLsFr0=vr0=vr0dc8meaabaqaciaacaGaaeqabaqabeGadaaakeaacqWGWbaCcqGGNaWjdaWgaaWcbaGaemyAaKMaemOAaOgabeaakiabg2da9maalaaabaGaemiCaa3aaSbaaSqaaiabdMgaPjabdQgaQbqabaGccqGHsislcqWGTbqBcqWGLbqzcqWGHbqycqWGUbGBcqGGOaakcqWGWbaCdaWgaaWcbaGaemyAaKMaemymaedabeaakiabcYcaSiabc6caUiabc6caUiabc6caUiabcYcaSiabdchaWnaaBaaaleaacqWGPbqAcqWGUbGBaeqaaOGaeiykaKcabaGaem4CamNaemizaqMaeiikaGIaemiCaa3aaSbaaSqaaiabdgdaXiabdQgaQbqabaGccqGGSaalcqGGUaGlcqGGUaGlcqGGUaGlcqGGSaalcqWGWbaCdaWgaaWcbaGaemOyaiMaemOAaOgabeaakiabcMcaPaaacqGGUaGlaaa@5DED@

We plotted each of the series *P*'_*i1 *_,...,*P*'_*in *_versus the series of standard deviations *s*_*j*_,...*s*_*n *_where *s*_*j *_= *s*_*d *_(*P*_1*j*_,...,*P*_*bj*_) in six qq-plots. The x-coordinates (*s*_*j*_) are equal in all the plots and allow avoiding assumptions about the distribution of the p-values.

A regression line, based on the standardized p-values between the first and third quartile is fitted to each qq-plot. We chose a relative cut-off at -0.9 (×S.d.) by inspection of all comparisons and transformed the cut-offs back to the original scales so that it can be compared to the original p-values.

## List of abbreviations

*ε*_*p *_– Error term for protein *p*

2D – 2D electrophoresis

A – 1/2 log_2 _(Cy5 × Cy3)

ACN – Acetonitril

AGC – Automated gain control

ALS – Amyotrophic Lateral Sclerosis

BVA – Biological Variance Analysis

cDNA – Complementary DNA

CHAPS – 3-[(3-Cholamidopropyl)Dimethyl-Ammonio]-1-Propanesulfonate

Ctl – Control

Cy2 – Cyanine

Cy3 – Indocarbocyanine

Cy5 – Indodicarbocyanine

Da – Dalton

DEPPS – Differential Expression in Predefined Protein *Sets*

DIGE – Difference in gel electrophoresis

DNA – deoxyribonucleic acid

DTT – Dithiothreitol

*DvC *– Comparison between Dysk and Ctl treated animals

*DvL *– Comparison between Dysk and Ldopa treated animals

*DvM *– Comparison between Dysk and Mptp treated animals

Dysk – Treatment with MPTP and L-DOPA leading to dyskinesia

ESI – Electro spray ionization

*F *– Effect of the fluorescent dye

FDR – False discovery rate (FDR* = n*/n)

GO – Gene Ontology

GSEA – Gene Set Enrichment Analysis

HAc – Acetic acid

IEF – Isoelectric focusing

IPG – Immobilized pH gradient

L-DOPA – Levodopa

Ldopa – Treatment with Levodopa

log_2 _– Logarithm with base 2

*LvC *– Comparison between Ldopa and Ctl treated animals

*LvM *– Comparison between Ldopa and Mptp treated animals

*M *– log_2_(*Cy5*/*Cy3*)

*m *– log_2_(*Cy5*/*Cy2*) and log_2_(*Cy3*/*Cy2*)

*MCy5 *– log_2_*Cy3/*(log_2_*Cy5*+ log_2_*Cy3*+ log_2_*Cy2*)/3

*M*Cy3 – log_2_*Cy5*/(log_2_*Cy5*+ log_2_*Cy3*+ log_2_*Cy2*)/3

*M*Cy2 – log_2_*Cy2*/(log_2_*Cy5*+ log_2_*Cy3*+ log_2_*Cy2*)/3

MPTP – 1-methyl-4-phenyl-1,2,3,6-tetrahydropyridine.

Mptp – Treatment with MPTP

MS – Mass Spectrometry

MS/MS – Tandem mass spectrometry or Mass spectrometry/Mass spectrometry

MSN – Medium spiny neurons

*MvC *– Comparison between Mptp and Ctl treated animals

NCBI – National Center for Biotechnology Information

NH_4_HCO_3 _– Ammonium bicarbonate

pI – Isoelectric point

Qq-plot – Quantile-quantile plot

S.d. – Standard deviation

SDS – Sodium dodecyl sulfate

SEM – Standard error of the mean

TCA – Tricarboxylic acid

TFA – Trifluoroacetic acid

Tris-HCl – 2-Amino-2-(hydroxymethyl)-1,3-propanediol, hydrochloride

Vsn – Variance stabilization normalization

Xcorr – Charge state vs. cross-correlation number

## Authors' contributions

All authors conceived and designed the experiments. AC and EB performed the *in vivo *experiments. EB realized the dissection. BS, HA, KS and MS achieved the *in vitro *processing of dissected tissues. BS did the image analysis and spot matching. KS and MS performed the protein identification and BS did the protein classification. PEA supervised the proteomic sample preparations, analyses, and identifications. IL and KK performed the analysis of the data. KK, IL and BS drafted the manuscript with significant contributions from all authors. Principal investigators, PEA, EB and ARC, contributed with funding. All authors read and approved the final manuscript.

## References

[B1] Alban A, David SO, Bjorkesten L, Andersson C, Sloge E, Lewis S, Currie I (2003). A novel experimental design for comparative two-dimensional gel analysis: two-dimensional difference gel electrophoresis incorporating a pooled internal standard. Proteomics.

[B2] Unlu M, Morgan ME, Minden JS (1997). Difference gel electrophoresis: a single gel method for detecting changes in protein extracts. Electrophoresis.

[B3] Dudoit S, Yang YH, Parmigiani G, Garrett ES, Irizarry RA, Zeger SL (2002). Bioconductor R packages for exploratory analysis and normalization of cDNA microarray data in:The Analysis of Gene Expression Data: Methods and Software.

[B4] Huber W, von Heydebreck A, Sultmann H, Poustka A, Vingron M (2002). Variance stabilization applied to microarray data calibration and to the quantification of differential expression. Bioinformatics.

[B5] Lönnstedt I, Speed T (2002). Replicated microarray data. Stat Sinica.

[B6] Smyth GK (2004). Linear models and empirical Bayes methods for assessing differential expression in microarray experiments. Stat Appl Genet Mol Biol.

[B7] Smyth GK, Speed T (2003). Normalization of cDNA microarray data. Methods.

[B8] Yang YH, Dudoit S, Luu P, Lin DM, Peng V, Ngai J, Speed TP (2002). Normalization for cDNA microarray data: a robust composite method addressing single and multiple slide systematic variation. Nucleic Acids Res.

[B9] Fodor IK, Nelson DO, Alegria-Hartman M, Robbins K, Langlois RG, Turteltaub KW, Corzett TH, McCutchen-Maloney SL (2005). Statistical challenges in the analysis of two-dimensional difference gel electrophoresis experiments using DeCyder. Bioinformatics.

[B10] Kreil DP, Karp NA, Lilley KS (2004). DNA microarray normalization methods can remove bias from differential protein expression analysis of 2D difference gel electrophoresis results. Bioinformatics.

[B11] Karp NA, Spencer M, Lindsay H, O'Dell K, Lilley KS (2005). Impact of replicate types on proteomic expression analysis. J Proteome Res.

[B12] Rowell C, Carpenter M, Lamartiniere CA (2005). Modeling biological variability in 2-D gel proteomic carcinogenesis experiments. J Proteome Res.

[B13] Alm H, Scholz B, Fischer C, Kultima K, Viberg H, Eriksson P, Dencker L, Stigson M (2006). Proteomic evaluation of neonatal exposure to 2,2 ,4,4 ,5-pentabromodiphenyl ether. Environ Health Perspect.

[B14] Subramanian A, Tamayo P, Mootha VK, Mukherjee S, Ebert BL, Gillette MA, Paulovich A, Pomeroy SL, Golub TR, Lander ES, Mesirov JP (2005). Gene set enrichment analysis: a knowledge-based approach for interpreting genome-wide expression profiles. Proc Natl Acad Sci U S A.

[B15] Tian L, Greenberg SA, Kong SW, Altschuler J, Kohane IS, Park PJ (2005). Discovering statistically significant pathways in expression profiling studies. Proc Natl Acad Sci U S A.

[B16] Ahlskog JE, Muenter MD (2001). Frequency of levodopa-related dyskinesias and motor fluctuations as estimated from the cumulative literature. Mov Disord.

[B17] Jenner P (2002). Pharmacology of dopamine agonists in the treatment of Parkinson's disease. Neurology.

[B18] Team RDC (2005). R: A language and environment for statistical computing.

[B19] Guigoni C, Li Q, Aubert I, Dovero S, Bioulac BH, Bloch B, Crossman AR, Gross CE, Bezard E (2005). Involvement of Sensorimotor, Limbic, and Associative Basal Ganglia Domains in L-3,4-Dihydroxyphenylalanine-Induced Dyskinesia. J Neurosci.

[B20] Hirata Y, Kiuchi K, Nagatsu T (2001). Manganese mimics the action of 1-methyl-4-phenylpyridinium ion, a dopaminergic neurotoxin, in rat striatal tissue slices. Neurosci Lett.

[B21] Rollema H, Kuhr WG, Kranenborg G, De Vries J, Van den Berg C (1988). MPP+-induced efflux of dopamine and lactate from rat striatum have similar time courses as shown by in vivo brain dialysis. J Pharmacol Exp Ther.

[B22] Fountoulakis M, Kossida S (2006). Proteomics-driven progress in neurodegeneration research. Electrophoresis.

[B23] Crosby AH (2003). Disruption of cellular transport: a common cause of neurodegeneration?. The Lancet Neurology.

[B24] Avila J, Lucas JJ, Perez M, Hernandez F (2004). Role of Tau Protein in Both Physiological and Pathological Conditions. Physiol Rev.

[B25] Cappelletti G, Surrey T, Maci R (2005). The parkinsonism producing neurotoxin MPP+ affects microtubule dynamics by acting as a destabilising factor. FEBS Letters.

[B26] Ren Y, Liu W, Jiang H, Jiang Q, Feng J (2005). Selective Vulnerability of Dopaminergic Neurons to Microtubule Depolymerization. J Biol Chem.

[B27] Ingham CA, Hood SH, Taggart P, Arbuthnott GW (1998). Plasticity of synapses in the rat neostriatum after unilateral lesion of the nigrostriatal dopaminergic pathway. J Neurosci.

[B28] Stephens B, Mueller AJ, Shering AF, Hood SH, Taggart P, Arbuthnott GW, Bell JE, Kilford L, Kingsbury AE, Daniel SE, Ingham CA (2005). Evidence of a breakdown of corticostriatal connections in Parkinson's disease. Neuroscience.

[B29] Gustafsson JS, Ceasar R, Glasbey CA, Blomberg A, Rudemo M (2004). Statistical exploration of variation in quantitative two-dimensional gel electrophoresis data. Proteomics.

[B30] Wheelock AM, Buckpitt AR (2005). Software-induced variance in two-dimensional gel electrophoresis image analysis. Electrophoresis.

[B31] Wheelock AM, Goto S (2006). Effects of post-electrophoretic analysis on variance in gel-based proteomics. Expert Rev Proteomics.

[B32] Cleveland WS, Grosse E, Shyu WM, Chambers JM, Hastie TJ (1992). Statistical Models in S. Local regression models.

[B33] Karp NA, Lilley KS (2005). Maximising sensitivity for detecting changes in protein expression: experimental design using minimal CyDyes. Proteomics.

[B34] Kerr MK, Churchill GA (2001). Experimental design for gene expression microarrays. Biostatistics.

[B35] Yang YH, Speed T (2002). Design issues for cDNA microarray experiments. Nat Rev Genet.

[B36] Bezard E, Dovero S, Prunier C, Ravenscroft P, Chalon S, Guilloteau D, Crossman AR, Bioulac B, Brotchie JM, Gross CE (2001). Relationship between the Appearance of Symptoms and the Level of Nigrostriatal Degeneration in a Progressive 1-Methyl-4-Phenyl-1,2,3,6-Tetrahydropyridine-Lesioned Macaque Model of Parkinson's Disease. J Neurosci.

[B37] Aubert I, Guigoni C, Hakansson K, Li Q, Dovero S, Barthe N, Bioulac BH, Gross CE, Fisone G, Bloch B, Bezard E (2005). Increased D1 dopamine receptor signaling in levodopa-induced dyskinesia. Ann Neurol.

[B38] Aubert I, Guigoni C, Li Q, Dovero S, Bioulac BH, Gross CE, Crossman AR, Bloch B, Bezard E (2006). Enhanced preproenkephalin-B-derived opioid transmission in striatum and subthalamic nucleus converges upon pallidus internalis in L-dopa induced dyskinesia. Biol Psychiatr.

[B39] Bezard E, Ferry S, Mach U, Stark H, Leriche L, Boraud T, Gross C, Sokoloff P (2003). Attenuation of levodopa-induced dyskinesia by normalizing dopamine D3 receptor function. Nat Med.

[B40] Bezard E, Gross CE, Qin L, Gurevich VV, Benovic JL, Gurevich EV (2005). L-DOPA reverses the MPTP-induced elevation of the arrestin2 and GRK6 expression and enhanced ERK activation in monkey brain. Neurobiol Dis.

[B41] Guigoni C, Dovero S, Aubert I, Li Q, Bioulac BH, Bloch B, Gurevich EV, Gross CE, Bezard E (2005). Levodopa-induced dyskinesia in MPTP-treated macaques is not dependent on the extent and pattern of nigrostrial lesioning. Eur J Neurosci.

[B42] Gene Ontology Consortium (2006). The Gene Ontology (GO) project in 2006. Nucl Acids Res.

[B43] Bolstad BM, Irizarry RA, Astrand M, Speed TP (2003). A comparison of normalization methods for high density oligonucleotide array data based on variance and bias. Bioinformatics.

[B44] Smyth GK, Michaud J, Scott HS (2005). Use of within-array replicate spots for assessing differential expression in microarray experiments. Bioinformatics.

